# Medicine–food homology bioactives in Parkinson’s disease: multi-target oxidative-stress modulation and translation to dietary supplements

**DOI:** 10.3389/fnut.2025.1677749

**Published:** 2025-11-21

**Authors:** Meng Wang, Yizhu Zhang, Qiong Wu, Sijia Ma, Chao Wang, Jiajia Sang

**Affiliations:** 1 Institute of Basic Theory of Chinese Medicine, China Academy of Chinese Medical Sciences, Beijing, China; 2 School of Integrated Chinese and Western Medicine, Nanjing University of Chinese Medicine, Nanjing, China; 3 Jiangsu Provincial Hospital of Traditional Chinese Medicine, Nanjing, China

**Keywords:** Parkinson’s disease, medicine–food homology, oxidative stress, Nrf2/ARE, NF-κB, PI3K/AKT, dietary supplements

## Abstract

**Background:**

No proven disease-modifying therapy exists for Parkinson’s disease (PD), and prior single-target antioxidants have shown limited, unsustained benefits, highlighting the need for safe multi-target strategies.

**Objective:**

To synthesize how medicine–food homology (MFH) compounds from Traditional Chinese Medicine (TCM)—polysaccharides, saponins/triterpenoids, polyphenols, carotenoids, and aromatic phenylpropanoids—modulate oxidative stress and PD-related neurodegeneration, and to outline formulation routes toward dietary-supplement development.

**Methods:**

We searched PubMed, Web of Science Core Collection, Embase (Ovid), and the Cochrane Library from inception through August 1, 2025 with prespecified concept blocks (“Parkinson’s disease,” “oxidative stress,” Nrf2/ARE, NF-κB, PI3K/Akt, autophagy, and MFH terms). English-language in-vitro, invertebrate, and PD-specific rodent studies, selected epidemiology, and formulation/dose/regulatory reports were narratively appraised; no meta-analysis or tool-based risk-of-bias scoring was performed.

**Results:**

MFH compounds converge on Nrf2/ARE activation, NF-κB suppression, autophagy promotion, and mitochondrial stabilization; nano-/micro-delivery may improve bioavailability and brain exposure in preclinical models. Evidence is predominantly preclinical, with heterogeneous methods and sparse PD-specific randomized trials; epidemiologic signals are suggestive but non-causal. PD-specific oxidative stress arises from dopamine auto-oxidation, neuromelanin–iron catalysis, and complex-I hypofunction; Latest studies further bind these to ferroptosis-linked lipid peroxidation. Clinical evidence remains sparse and PK-limited for MFH actives (e.g., curcumin, EGCG); dose–response, safety monitoring (including liver signals for catechins), and regulatory constraints frame translation.

**Conclusion:**

MFH compounds are promising, hypothesis-generating candidates for adjunctive nutrition in PD, pending clinical dose–response and long-term safety validation. No clinical efficacy has been established.

## 1 Introduction

### 1.1 Clinical characteristics and therapeutic limitations of Parkinson’s disease

Parkinson’s disease (PD) is the second most prevalent neurodegenerative disorder worldwide, characterized clinically by resting tremor, bradykinesia, rigidity of the limbs and trunk, and postural instability (1). Multiple recent sources project a continued rise in PD prevalence with population aging, although absolute estimates vary by modeling assumptions and data inputs (2, 3). Current management of PD is dominated by oral pharmacotherapy, principally levodopa–carbidopa combinations; patients with concomitant cognitive impairment often receive rivastigmine (4). However, all existing agents exhibit fluctuating efficacy and notable adverse effects. Long-term dopaminergic replacement frequently leads to “end-of-dose” deterioration and motor complications (5, 6), while rivastigmine may provoke nausea, vomiting, and tremor (7). Moreover, chronic use of these medications commonly induces gastrointestinal disturbances (8, 9) and further movement disorders (10). These limitations underscore the urgent need for novel therapeutic strategies to slow disease progression and improve patient quality of life. See Evidence Capsule 6.7 for 2024–2025 multi-source updates (GBD-based projections, regional meta-analyses, and narrative syntheses) that triangulate these trends and explain variability across methods and regions.

### 1.2 Advantages of traditional Chinese medicine in multi-target regulation and holistic therapy

The progressive loss of dopaminergic neurons in the substantia nigra pars compacta (SNpc) constitutes the principal pathological hallmark of PD (11), with oxidative stress recognized as a key driving mechanism. Studies (12–14) have demonstrated that targeting oxidative stress can effectively ameliorate PD symptoms and delay disease progression. Current pharmacological interventions for oxidative stress in the nervous system—such as edaravone (15, 16) and N-acetylcysteine (16, 17)—act on single targets and employ relatively narrow mechanisms, and are associated with dose-limiting adverse effects and loss-of-benefit phenomena (e.g., end-of-dose deterioration and motor complications), rather than durable disease modification (18). In contrast, traditional Chinese medicine (TCM) embodies the “medicinal-food homology”(MFH) concept (19) and leverages multi-component formulations (20) to address multifactorial and multi-target pathologies such as PD. Medicinal-food homologous herbs—e.g., *Lycium barbarum* and *Poria cocos*—simultaneously scavenge free radicals, activate the Nrf2/ARE pathway, inhibit neuroinflammation, and stabilize mitochondrial function (21, 22). Moreover, TCM compound formulations exert synergistic, multi-target therapeutic effects with favorable long-term tolerability, thereby overcoming the limitations of single-target agents like edaravone and N-acetylcysteine. Compared to conventional pharmaceuticals, natural bioactive constituents from TCM can mitigate oxidative stress in the nervous system through multiple pathways while exhibiting a lower incidence of adverse reactions (14, 23). Notwithstanding these advantages, evidence quality is heterogeneous. Batch-to-batch variability, potential herb–drug interactions (e.g., with levodopa or MAO-B inhibitors), and limited long-term safety data in PD populations warrant cautious interpretation and prospective clinical validation.

### 1.3 Article structure and research objectives

This review aims to fill a gap in the existing literature by systematically surveying the effects of various MFH herbs on oxidative stress and neurodegeneration in PD, with a particular focus on five major categories: polysaccharides, saponins/triterpenes, polyphenols, carotenoids, and aromatic phenylpropanoids. We will first introduce representative herbs from each category and their principal bioactive constituents—such as curcumin (CUR) and eugenol—detailing the latest mechanistic insights into free-radical scavenging, activation of the Nrf2/ARE pathway, inhibition of neuroinflammation, and stabilization of mitochondrial function. Next, we will explore strategies for multi-component synergy and formula design, as well as feasible approaches to translate these findings into functional dietary supplements. Finally, we will assess current bottlenecks in clinical translation and propose future research directions, with the goal of providing a theoretical foundation and a systematic reference for TCM-based adjuvant interventions in PD and the development of related functional foods. This review specifically addresses gaps in prior syntheses by (i) mapping compound–target–pathway linkages for oxidative stress in PD-relevant models rather than mixed neurodegeneration, and (ii) integrating formulation science (nano/micro-delivery, dosing anchors, and regulatory context) to outline translational paths toward standardized dietary supplements.

### 1.4 PD-specific oxidative stress features

**Figure 1:**
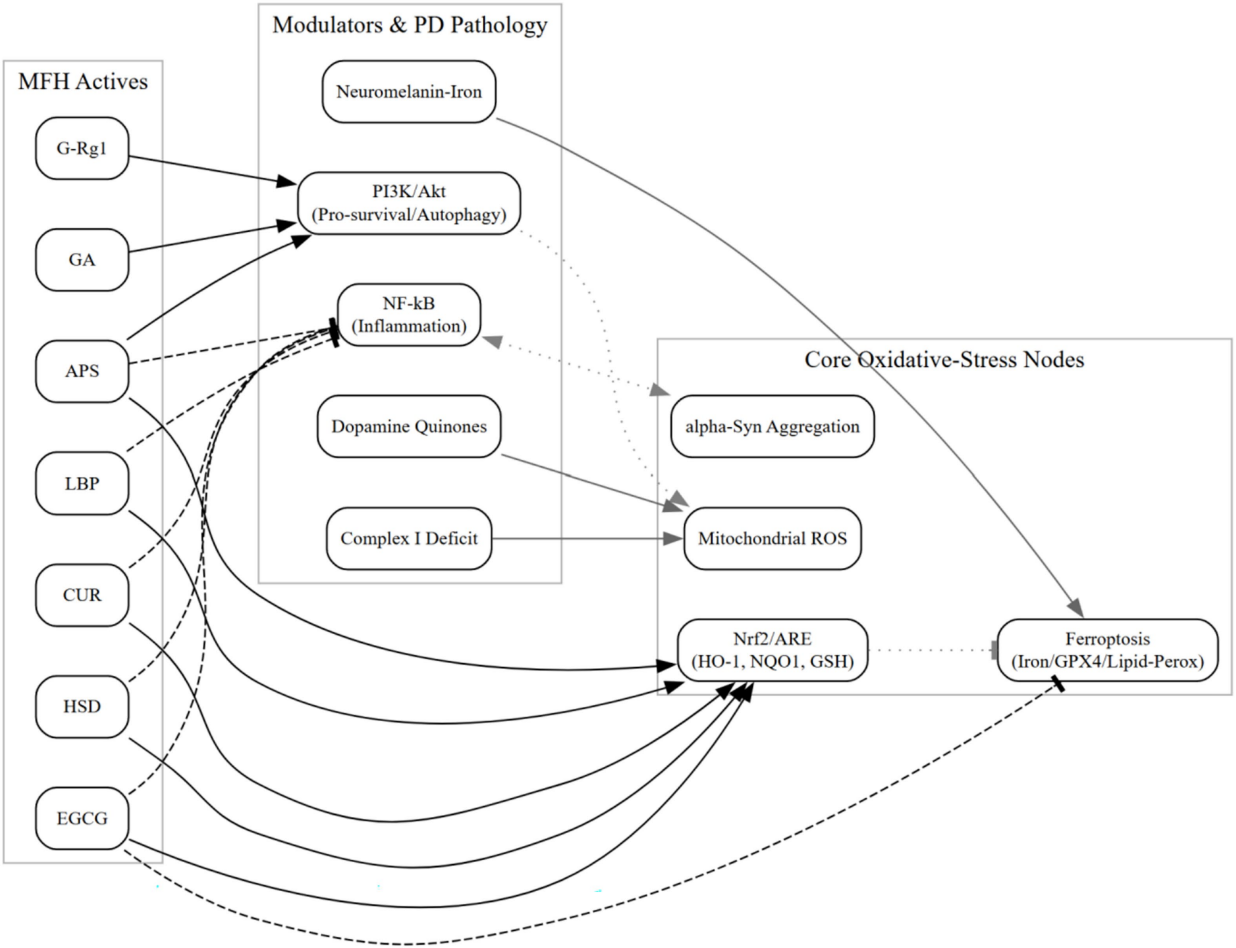
Schematizes MFH actives targeting Nrf2 activation, mitochondrial ROS restraint, ferroptosis inhibition (iron handling and lipid-peroxidation control), and α-synuclein aggregation checkpoints, with cross-talk to PI3K/Akt and NF-κB. Edges summarize PD-relevant preclinical evidence; no clinical efficacy is implied (see Sections 6.1 and 6.6).

PD-specific redox stress converges on (i) cytosolic dopamine auto-oxidation with redox-cycling quinones that generate H_2_O_2_ and catechol quinones toxic to SNpc neurons; (ii) neuromelanin–iron interactions catalyzing Fenton chemistry and amplifying lipid peroxidation/ferroptosis; and (iii) complex-I hypofunction with bioenergetic fragility and ROS spillover. Recent datasets refine in-vivo dopamine quinone chemistry, quantify neuromelanin-bound iron catalysis, and stratify idiopathic PD by complex-I deficiency, strengthening that oxidative stress is disease-relevant (not generic) and mechanistically linked to ferroptosis. See Section 6.1 for capsule updates and Figure 1 for a schematic.

### 1.5 Genetic forms intersecting oxidative stress (GBA1, LRRK2, PINK1/PARKIN, DJ-1)

Monogenic PD further anchors oxidative stress: PINK1/Parkin loss impairs mitophagy and elevates ROS; DJ-1 acts as a redox-sensitive chaperone; LRRK2 variants perturb mitochondrial dynamics; GBA1 insufficiency compromises lysosomal clearance and secondarily heightens oxidative injury. These nodes outline plausible interfaces for MFH actives, ferroptosis restraint and mitochondrial support (hesperidin)—but direct genotype-informed human data are lacking and remain hypothesis-generating. Further genotype–oxidative intersections (GBA1, LRRK2, PINK1/PARKIN, DJ-1) and plausible MFH interfaces are summarized in Section 6.2.

### 1.6 Literature search and eligibility

We conducted a structured narrative search of PubMed, Web of Science Core Collection, Embase (Ovid), and the Cochrane Library from database inception through August 1, 2025. Predefined concept blocks combined PD with oxidative-stress pathways (e.g., “oxidative stress,” Nrf2/ARE, NF-κB, PI3K/Akt, autophagy) and medicinal–food homologous Traditional Chinese Medicine terms (e.g., polysaccharide, saponin/triterpen, polyphenol, carotenoid, eugenol, hesperidin, and source herbs such as Lycium, Poria, Panax, Glycyrrhiza). Searches were limited to English. We included primary *in vitro*, invertebrate, and PD-specific rodent studies, epidemiologic analyses, and formulation/delivery reports relevant to oxidative stress in PD; narrative reviews and non-PD basic studies were excluded unless providing mechanistic or translational rationale. No quantitative meta-analysis or formal tool-based risk-of-bias scoring was performed. Most included records were published in 2020–2025, reflecting field growth rather than a date-restricted criterion, and may introduce recency and publication biases. Full database-specific search strings are provided in Appendix A. Because this is a structured narrative synthesis without a preregistered protocol or quantitative pooling, we did not enumerate record counts or produce a PRISMA flow diagram.

## 2 Preclinical and mechanistic evidence of TCM active constituents in PD

We conducted a narrative appraisal of study-design features as reported in the included articles, noting the presence or absence of randomization, blinding, allocation concealment, sample-size justification, outcome-assessor masking, selective reporting, and funding or conflicts of interest. A formal checklist-based risk-of-bias assessment (e.g., SYRCLE or Cochrane RoB 2) was not performed.

We first summarize mechanistic evidence from non-PD models (e.g., ischemia–reperfusion, AD) as indirect evidence, acknowledging translational limitations; subsequent sections focus on PD-relevant models (MPTP, 6-OHDA). These cross-model mechanistic studies lay the groundwork for their application in PD. The following sections will focus specifically on PD-relevant models—such as MPTP and 6-OHDA in cell cultures and rodents—to systematically summarize the targeted effects of each major active constituent in PD.

### 2.1 Polysaccharides

#### 2.1.1 *Lycium barbarum*—*Lycium barbarum* polysaccharides

*Lycium barbarum* polysaccharides (LBP) is one of the primary active constituents of *Lycium barbarum*, exerting neuroprotective effects primarily through activation of the Nrf2/ARE antioxidant pathway (24) and inhibition of the NF-κB inflammatory pathway (25) to protect dopaminergic neurons. In the Nrf2/ARE pathway, LBP significantly increases the activity of superoxide dismutase (SOD) and glutathione peroxidase (GSH-Px), scavenges excessive reactive oxygen species (ROS), stabilizes mitochondrial membrane potential (MMP), and reduces cytochrome C release (26, 27). In the NF-κB pathway, it inhibits phosphorylation of IκBα, blocks the nuclear translocation of p65, and reduces the secretion of TNF-α and IL-1β, thereby alleviating neuroinflammation and delaying motor dysfunction (25, 28). In recent animal studies, Song et al. (29) demonstrated that in a silkworm model of PD, LBP significantly enhanced SOD and GSH-Px activity, scavenged excess ROS, stabilized MMP, and reduced cytochrome C release—effects closely associated with the Nrf2/HO-1 pathway. Cao et al. (30) found in an H₂O₂-induced PC12 oxidative neurotoxicity model and a CoCl₂-treated behavioral impairment rat model that intraperitoneal administration of LBP at 100 mg/kg d decreased ROS levels and improved spatial learning, memory deficits, and cognitive impairment in rats. Regarding the inhibition of the NF-κB pathway, most current studies of LBP focus on relieving oxidative stress and improving cognitive symptoms, while direct research on PD remains limited. For example, Gao et al. (31) found that in a 6-hydroxydopamine (6-OHDA)-induced PC12 cell apoptosis model, LBP significantly inhibited excessive NF-κB activation (p65 nuclear translocation) and downregulated neuronal nitric oxide synthase (nNOS) and inducible nitric oxide synthase (iNOS) expression, thereby reducing apoptosis. Zheng et al. (32) found that oral LBP downregulated mRNA and protein expression of TLR4, MyD88, and NF-κB, alleviated hippocampal inflammation and neural damage, and improved cognitive deficits in mice. [Note: this paragraph includes indirect mechanistic evidence from non-PD models; e.g., references (24, 26, 27)].

#### 2.1.2 *Astragalus membranaceus*—*Astragalus* polysaccharides

*Astragalus* polysaccharides (APS) alleviates oxidative stress in the nervous system primarily through the Nrf2/ARE and TLR4/MyD88/NF-κB pathways. It not only eliminates ROS, enhances SOD and GSH-Px activity, and reduces malondialdehyde (MDA) levels (33), but also inhibits the TLR4-mediated MyD88 cascade, decreases p65 nuclear translocation and downstream inflammatory factors, and simultaneously activates the PI3K/Akt pathway, increasing the Bcl-2/Bax ratio and inhibiting caspase-3-mediated apoptosis (23). Current studies on APS in the treatment of PD include the following: Tan et al. (34) reported that APS enhanced autophagy via the PI3K/AKT/mTOR pathway in an *in vitro* PD cell model, while also inhibiting Bad/Caspase-3 and increasing the Bcl-2/Bax ratio, thus exerting neuroprotective effects. In animal experiments, Shi and Ma (33) indicated that APS reduced MDA accumulation, restored the antioxidant function of SOD and GSH-Px, and lowered ROS levels, thereby relieving oxidative stress-induced neuronal injury in PD. [Note: this paragraph includes indirect mechanistic evidence from non-PD models; e.g., reference (33)].

### 2.2 Saponins/triterpenes

#### 2.2.1 *Panax ginseng*—ginsenoside Rg1

Ginsenoside Rg1 (G-Rg1) enhances neuronal survival by activating the PI3K/Akt anti-apoptotic signaling pathway (35). The underlying mechanism involves phosphorylated Akt upregulating Bcl-2 and inhibiting Bad and caspase-3 activity (36). Additionally, G-Rg1 promotes the expression of Beclin 1 and LC3 II via the AMPK/mTOR autophagy pathway, accelerating the clearance of harmful proteins, reducing oxidative stress sources and macromolecular oxidation, and facilitating Nrf2 nuclear translocation and antioxidant enzyme expression (37). Huang et al. (38) confirmed through *in vitro* experiments that Rg1 significantly increases the phosphorylation levels of PI3K and Akt, upregulates the anti-apoptotic protein Bcl-2, and inhibits the activity of Bad and caspase-3, thereby preventing hippocampal neuronal degeneration. Researchers (39) also found that treatment with G-Rg1 in an MPTP-induced mouse model restored the LC3 II/I ratio and Beclin-1 expression, promoted lysosome-mediated clearance of α-synuclein aggregates, and alleviated dopaminergic neuronal loss and motor deficits. [Note: this paragraph includes indirect mechanistic evidence from non-PD models; e.g., references (37, 38)].

#### 2.2.2 *Glycyrrhiza uralensis*—glycyrrhizic acid

Glycyrrhizic acid (GA) alleviates oxidative stress via two synergistic mechanisms: inhibition of the HMGB1/TLR4/NF-κB pathway and activation of the PI3K/Akt pathway. On one hand, it suppresses HMGB1 release and blocks the downstream TLR4 cascade, thereby reducing the production of inflammatory cytokines (40); on the other hand, it activates Akt, increases the Bcl-2/Bax ratio, inhibits caspase-3, and enhances GSH content, thereby protecting mitochondrial function (41). Current experiments suggest that GA has therapeutic potential in PD. For example, Kartik et al. (42) demonstrated that glycyrrhizic acid significantly improved motor deficits in MPTP-treated mice by enhancing autophagy, inhibiting nNOS/NO signaling, restoring SOD activity, and reducing MDA and inflammatory mediators. Zeng et al. (43) reviewed previous literature and found that GA not only alleviates neural injury by suppressing HMGB1/TLR4/NF-κB inflammatory signaling, but also relieves oxidative stress by restoring SOD and GSH-Px activity and reducing ROS and MDA levels. Through these pathways, GA improves both motor and cognitive functions in experimental animals. [Note: this paragraph includes indirect mechanistic evidence from non-PD models; e.g., references (40, 41)].

### 2.3 Polyphenols

#### 2.3.1 *Curcuma longa*—curcumin

Curcumin (CUR) reversibly inhibits MAO-B to reduce H₂O₂ production (44) and activates the Nrf2/ARE signaling pathway, upregulating HO-1 and NQO1 and scavenging ROS (45). It also inhibits NF-κB p65 nuclear translocation, reducing COX-2 and IL-6 expression, thereby mitigating oxidative and inflammatory damage at multiple targets (46). Current studies have focused on several aspects. For instance, Rathore et al. (47) found that CUR significantly restored motor coordination and antioxidant activity and improved mitochondrial function in a rotenone-induced PD mouse model. The mechanism involves p62–Keap1-mediated Nrf2 release, upregulation of HO-1 and NQO1, enhanced ROS clearance, and autophagy-mediated removal of damaged proteins. Xu et al. (48) reported that daily administration of 50 mg/kg curcumin (intraperitoneal) effectively prevented PD-like symptoms induced by rotenone in mice, likely due to its inhibition of microglial NF-κB-NLRP3 inflammasome activation and reduction of pro-inflammatory cytokines such as IL-1β and IL-18.

#### 2.3.2 *Camellia sinensis*—epigallocatechin gallate

Epigallocatechin gallate (EGCG) mitigates oxidative stress associated with neurological disorders via multiple pathways. It directly scavenges excessive ROS and reduces H₂O₂ generation, thereby lowering oxidative stress in neurons (49). It activates the Keap1/P62/Nrf2/ARE signaling pathway, upregulates antioxidant enzymes such as HO-1 and NQO1, and enhances intracellular glutathione (GSH) and NQO1 levels (50). EGCG also inhibits NF-κB p65 nuclear translocation, reducing the expression of COX-2 and IL-6, and thereby attenuating oxidative and inflammatory damage to dopaminergic neurons induced by 6-OHDA (51). In various models of neurodegenerative diseases, EGCG modulates signaling pathways such as PI3K/Akt and MAPK, suppresses neuroinflammation, and improves cognitive and motor function, demonstrating its multi-target neuroprotective synergy. Xia et al. (52) found that EGCG treatment in a PINK1-mutant *Drosophila* PD model significantly reduced brain iron accumulation and MDA levels, while increasing SOD and GSH-Px activity, reducing ROS production, and inhibiting iron-dependent cell death. Xu et al. (49) showed that in a 6-OHDA-induced N27 cell model pretreated with EGCG, intracellular ROS and H₂O₂ levels were significantly reduced, Nrf2 nuclear translocation was promoted, and HO-1 and NQO1 expression was increased, thereby enhancing antioxidant enzyme activity and reducing oxidative damage and apoptosis. In a mouse PD model induced by α-synuclein preformed fibrils (PFFs), EGCG treatment decreased TNF-α and IL-6 expression, reduced pathological aggregation, increased survival of dopaminergic neurons in the substantia nigra, and improved both motor and anxiety-like behaviors (53). Shen et al. (54) reported that EGCG restored motor function and protected TH^+^ neurons in the SNpc region in an MPTP-induced PD model in C57BL/6 mice. Mechanistic studies showed that EGCG activated PI3K/Akt pro-survival signaling and suppressed the MAPK pathway, thereby reducing neuroinflammation and neuronal apoptosis. Amin et al. (55) further demonstrated that in models of PD and other neurodegenerative diseases, EGCG exerts neuroprotective effects via NF-κB signaling inhibition by blocking IκBα phosphorylation and suppressing p65 nuclear translocation, thus downregulating pro-inflammatory cytokines such as TNF-α and IL-1β and reducing dopaminergic neuronal loss and motor dysfunction. [Note: this paragraph includes indirect mechanistic evidence from non-PD models; e.g., references (50, 51)].

### 2.4 Aromatic compounds (phenylpropanoids)

#### 2.4.1 Clove—eugenol

Eugenol restores or enhances SOD, GSH-Px, and reduced glutathione (GSH) levels, thereby improving ROS scavenging capacity. It also alleviates oxidative damage to cellular membranes by reducing malondialdehyde (MDA) levels (56). These are the two primary mechanisms through which eugenol mitigates oxidative stress. In animal models, Vora et al. (57) pretreated MPTP-induced C57BL/6 mice with eugenol for 7 days and found that it improved motor deficits such as gait disturbances, restored brain GSH levels, and reduced MDA levels, thereby exerting neuroprotective effects. In a 6-OHDA-induced unilateral rat PD model, eugenol reduced oxidative-stress markers; when co-administered with levodopa, it further increased reduced glutathione (GSH), indicating an additive redox effect (58).

#### 2.4.2 *Citrus reticulata*—hesperidin

Hesperidin (HSD) combats oxidative stress primarily by activating the Nrf2/ARE pathway and inhibiting NF-κB signaling. It promotes Nrf2 nuclear translocation and upregulates HO-1 and NQO1 (59); in addition, it blocks p65 nuclear translocation by inhibiting IκBα phosphorylation, thereby scavenging ROS and preserving mitochondrial function (60). In animal studies, five-week oral administration of HSD in rat PD models significantly improved performance in rotarod and spontaneous movement tests. It suppressed IκBα phosphorylation and p65 nuclear translocation in the striatum and substantia nigra, downregulated COX-2 and IL-6 levels, reduced MDA levels, and restored SOD and GSH-Px activity (61). Adedara et al. (62) found that in an MPTP-induced *Drosophila* PD model, HSD treatment for 7 days lowered MDA and protein carbonyl levels and corrected the dysregulated Keap1/Nrf2 mRNA expression, indicating activation of the Nrf2/ARE pathway. [Note: this paragraph includes indirect mechanistic evidence from non-PD models; e.g., references (59, 60)].

### 2.5 Carotenoids

#### 2.5.1 *Hippophae rhamnoides*—β-carotene

β-carotene (β-Car) mitigates oxidative stress and neuroinflammation by reducing MDA and NF-κB p65 levels, while enhancing the activity of SOD, GSH, and GSH-Px (63). It also decreases ROS levels and restores antioxidant enzyme activity (64). Recent studies have mainly focused on ROS modulation and clinical relevance. For example, Chaves et al. (65) found that in a *Drosophila* PD model, β-Car nanoparticles significantly increased SOD and GSH-Px activity, decreased ROS and MDA levels, improved motor performance, and promoted dopaminergic neuron survival. Clinically, Su et al. (66) conducted cross-sectional and cohort studies revealing a significant inverse association between β-Car intake and both PD prevalence and all-cause mortality in the general population. [Note: this paragraph includes indirect mechanistic evidence from non-PD models; e.g., references (63, 64)].

#### 2.5.2 *Lycium barbarum*—lutein

Lutein exerts multifaceted neuroprotective effects. On one hand, it promotes Nrf2 nuclear translocation and binding to ARE elements, upregulating HO-1 and NQO1 expression and enhancing antioxidant enzyme activity to clear ROS (67). On the other hand, it blocks IKKβ-mediated phosphorylation of IκBα and inhibits NF-κB p65 nuclear translocation, thereby downregulating pro-inflammatory cytokines such as TNF-α and IL-1β, reducing neuroinflammation, and preserving mitochondrial function—ultimately alleviating both oxidative and inflammatory neuronal damage (68). However, in the past 5 years, there has been a lack of studies exploring lutein’s role in PD using molecular mechanistic approaches or mammalian/invertebrate models. Only Fernandes et al. (69) have reported that lutein treatment in a *Drosophila* PD model improved climbing ability and survival rate and restored brain dopamine levels, tyrosine hydroxylase activity, and SOD, catalase (CAT), and glutathione S-transferase (GST) levels, suggesting phenotypic improvement through antioxidant mechanisms.

### 2.6 Others

Several other medicine-food homologous compounds also exhibit neuroprotective effects by alleviating oxidative stress, although none have been validated through PD-specific animal models. For instance, pachymaran (PCP), the major constituent of *Poria cocos*, activates the Nrf2/ARE pathway, upregulates HO-1 and NQO1 gene expression, enhances intrinsic antioxidant capacity, and suppresses TLR4/NF-κB signaling to reduce inflammatory mediator release (70). It also increases intracellular GSH, further reduces ROS, and helps maintain mitochondrial function and neuronal survival (71). *Ganoderma* triterpenes (GLTs), active components of *Ganoderma lucidum*, were shown to reduce neuronal apoptosis, lower ROS and MDA levels, and enhance mitochondrial function in AD mouse models (72). GLTs also upregulate SOD and CAT expression, decrease MDA levels, and activate the Nrf2/HO-1 pathway to exert antioxidative neuroprotection (73). In Aβ25–35-induced AD rat models, hawthorn flavonoids (HF) significantly increased HO-1 and NQO1, reduced MDA, and enhanced SOD activity, thereby reducing oxidative stress and neurodegeneration (74). HF also decreased ROS and pro-inflammatory cytokines, stabilized mitochondrial membrane potential, and improved cognition and behavior (75). In addition, some active ingredients such as eugenol possess multiple neuroprotective mechanisms. Eugenol induces Nrf2 nuclear translocation via the Nrf2/ARE pathway, upregulates HO-1 and NQO1, and prevents neuronal injury, improving neurological symptoms such as epilepsy (76). Eugenol also alleviates neuroinflammation by inhibiting IκBα phosphorylation and blocking p65 nuclear translocation, enhances membrane fluidity, and increases neuronal resilience, thereby mitigating aluminum-induced neurotoxicity, oxidative stress, neuronal loss, and reactive astrocytosis (77). While these pathways have not yet been studied in PD-specific models, other herbal compounds have shown efficacy through similar mechanisms (24, 33). In summary, although relatively few studies have explored PD treatment through oxidative stress modulation over the past 5 years, the established neuroprotective and mechanistic similarities justify further investigation.

## 3 Key mechanistic pathways and synergistic actions

**Figure 2:**
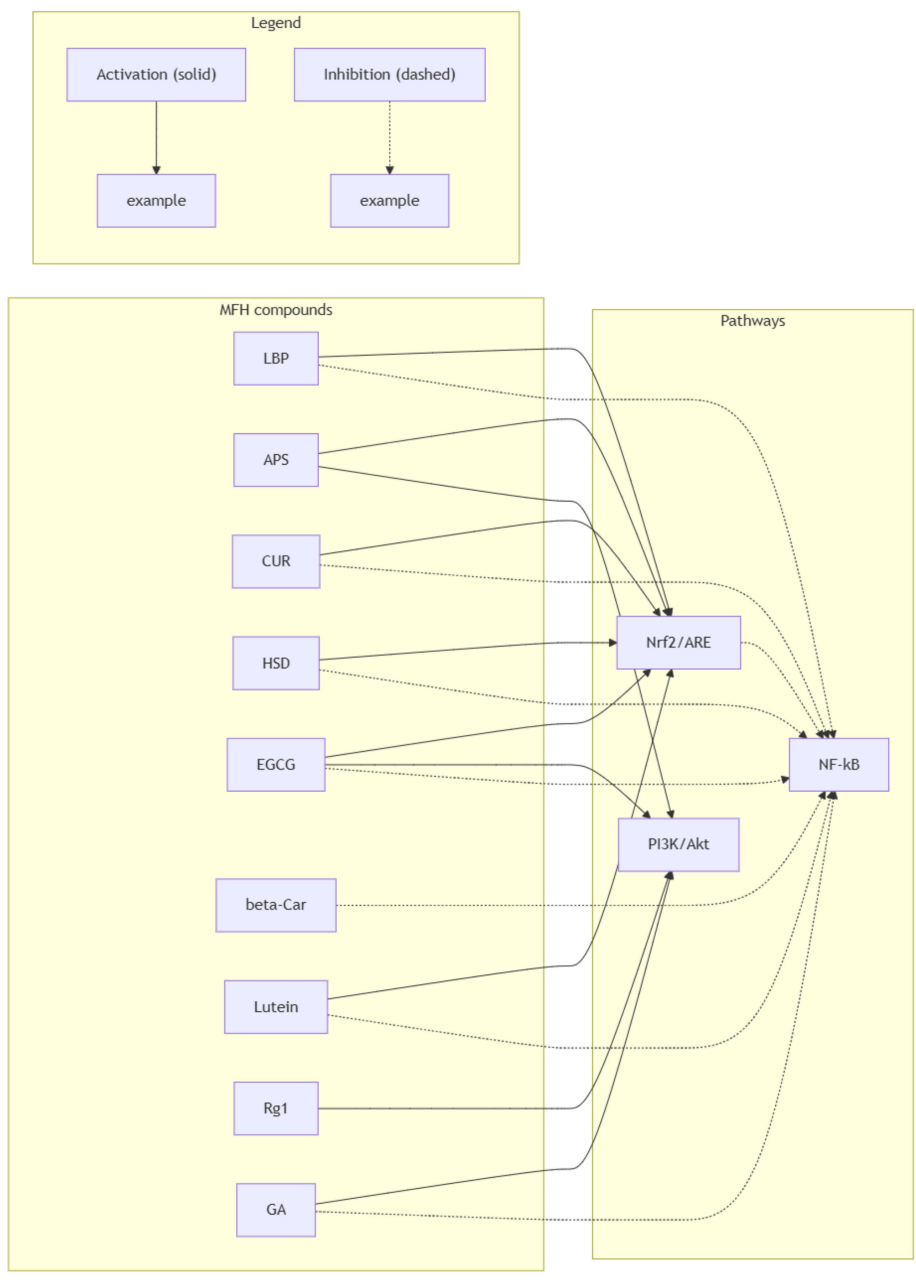
Cross-talk between Nrf2/ARE, PI3K/Akt and NF-κB targeted by representative MFH compounds relevant to Parkinson’s disease. Solid arrows denote activation; dashed arrows denote inhibition. Edges summarize predominantly preclinical evidence in PD-relevant models (with selective mechanistic support from non-PD models as noted in the text) and are schematic, not implying clinical efficacy.

In clinical application, TCM is often administered in multi-herbal formulations (78–80), which typically involve multiple herbs targeting the same signaling pathway (78, 81) or a single herb acting through multiple mechanisms for the same disease (82, 83). To orient the reader, Figure 2 schematizes the core oxidative-stress network, with compound-level cross-references in Appendix B: Supplementary Table 1. We therefore proceed to the cross-talk and potential synergy among Nrf2/ARE, PI3K/Akt, and NF-κB; a concise pathway-level summary linking representative MFH actives to these three axes is provided in Appendix B: Supplementary Table 2.

### 3.1 Activation of the Nrf2/ARE pathway

The Nrf2/ARE pathway exerts cross-regulatory control over neuroinflammation and mitochondrial function through multiple mechanisms (84). For instance, Nrf2 can inhibit NF-κB-mediated expression of pro-inflammatory cytokines such as TNF-α and IL-1β, making it a promising therapeutic target in neurodegenerative disorders (85). The Nrf2/ARE pathway itself has diverse regulatory routes (86, 87). Both LBP and APS promote the nuclear translocation of Nrf2, upregulate HO-1 and NQO1 expression, enhance ROS clearance, restore SOD and GSH-Px activity, and stabilize mitochondrial membrane potential (24, 33). CUR and EGCG have also been shown to activate Nrf2 in rotenone- or 6-OHDA-induced PD models, via direct Keap1 modification or p62-mediated Keap1 autophagic degradation, respectively—resulting in elevated antioxidant enzyme levels and significantly reduced oxidative damage in model animals (47, 52). Across models, Nrf2 activation intersects ferroptosis control via GPX4/iron handling and lipid-peroxidation readouts, aligning with PD-specific oxidative features noted in Sections 6.1 and 6.6.

### 3.2 PI3K/Akt signaling modulation

The PI3K/Akt signaling pathway serves as a key regulatory hub for cellular stress response, survival, and metabolism (88, 89). In PD models, dysregulation of this pathway exacerbates oxidative stress and apoptosis in dopaminergic neurons (90). G-Rg1, APS, GA, and EGCG are all capable of activating the PI3K/Akt pathway. G-Rg1, APS, and GA exhibit similar mechanisms: activating the pathway, promoting Akt phosphorylation, upregulating the Bcl-2/Bax ratio, and inhibiting caspase-3 activity to protect neurons and attenuate oxidative stress (23, 35, 36, 40). In MPTP-induced mice, EGCG not only activates PI3K/Akt survival signaling but also inhibits the MAPK pathway to reduce neuroinflammation and neuronal apoptosis (54). Furthermore, APS enhances autophagic activity through the PI3K/Akt–mTOR axis in 6-OHDA-treated PC12 cells (34).

### 3.3 NF-κB-mediated inflammation suppression

NF-κB is a transcription factor composed primarily of a p65/p50 heterodimer, extensively involved in immune and inflammatory regulation (91, 92). This classical signaling pathway has been repeatedly implicated in chronic inflammation and neurodegenerative disorders such as PD and is recognized as a key anti-inflammatory therapeutic target (93, 94). In PD models, LBP, EGCG, HSD, GA, and CUR have all been shown to inhibit NF-κB signaling. By blocking IκBα phosphorylation and preventing p65 nuclear translocation, these compounds downregulate pro-inflammatory cytokines, mitigate neuroinflammation, and improve behavioral outcomes (31, 43, 48, 55, 60). Both LBP and GA inhibit TLR4/NF-κB signaling (32, 43), thereby relieving oxidative stress and reducing inflammation and cellular injury.

Clearly, compared with monotherapy, herbal combinations may exert synergistic effects on PD by simultaneously acting on Nrf2/ARE, PI3K/Akt, and NF-κB pathways. The use of TCM formulas harnesses the complementary functions of multiple active compounds to relieve oxidative stress and inflammation, which constitutes a major mechanism underlying the preservation of dopaminergic neuron survival and function (95). Additionally, multi-target synergy (78) may help avoid the diminished efficacy and tolerance often seen with single-compound therapies during long-term intervention, thus enabling more sustainable and stable therapeutic outcomes (96). Anti-inflammatory NF-κB restraint may be potentiated by microbiota shifts reported for MFH polysaccharides/phenolics, suggesting an oxidative–inflammatory–gut axis relevant to PD (see Sections 6.6 and 4.4).

## 4 Feasibility of dietary supplementation

With growing public awareness of health management and diversification in the demand for “food-based therapies,” dietary supplements have become an important tool for daily health maintenance (97, 98). TCM resources classified as MFH offer both the safety profile of common food ingredients and the multi-target regulatory advantages of herbal medicine. Rich in natural bioactive components such as polysaccharides, saponins, triterpenes, polyphenols, and carotenoids, these herbs complement conventional pharmaceuticals (99). Numerous experimental and clinical studies (18, 100–102) indicate that converting these bioactives into standardized nanoemulsions, microemulsions, or encapsulated dietary supplements can not only enhance their antioxidative, anti-inflammatory, and neuroprotective effects in preclinical models; in selected preclinical models, co-administration with levodopa appeared to modulate levodopa response and adverse-effect readouts; clinical confirmation is lacking. This section elaborates on the definition and features of dietary supplements, specific needs of PD patients, current application status, and existing challenges—laying the foundation for the development of practical and science-based nutritional strategies.

### 4.1 Definition and application needs of dietary supplements in PD

Dietary supplements are defined as auxiliary nutritional agents derived from food-based ingredients or their extracts, formulated using technologies such as nanoemulsion, microemulsion, liposomal encapsulation, or microencapsulation to standardize and concentrate natural actives. These products occupy a niche between conventional food and prescription medications (103, 104). The pathogenesis of PD involves multiple mechanisms such as oxidative stress, neuroinflammation, and autophagy dysfunction. Long-term use of dopaminergic therapies like levodopa is often accompanied by wearing-off (end-of-dose) fluctuations and motor complications (5, 6). Dietary supplements, through their multi-target synergy, not only mitigate oxidative and inflammatory stress but may also reduce gastrointestinal side effects and dyskinesia risk, while offering neuroprotective, immunomodulatory, and microbiota-regulating benefits—fulfilling urgent unmet needs for adjunctive PD management (105, 106).

### 4.2 Formulation technologies and research advances: current dosage forms and doses

#### 4.2.1 Dosage forms

Advanced formulation techniques such as nanoemulsion (107), microemulsion (108), liposomes (109), and microencapsulation (110) have been widely applied to enhance the bioavailability and blood–brain barrier permeability of TCM-derived polysaccharides, saponins, triterpenes, polyphenols, and carotenoids (111, 112). These technologies have already been incorporated into PD treatment research. For example, Ramires Junior et al. (113) developed a porphyrin-assisted CUR nanoemulsion (NC) in a 6-OHDA-induced rat model and found that compared to free CUR, the NC formulation significantly improved motor performance, mitochondrial activity, and antioxidant parameters (MDA, SOD, GSH-Px). Imran et al. (114) optimized a silibinin (SLM) oil-in-water microemulsion using central composite design and demonstrated that intranasal administration significantly increased brain accumulation and improved motor function while suppressing neuroinflammation and oxidative stress in an MPTP model. Liu et al. (115) administered curcumin liposomes (BLIPO-CUR) camouflaged with NK cell membranes via the meningeal lymphatic vessels (MLVs) in PD mice and achieved targeted delivery to dopaminergic neurons, enabling ROS scavenging, motor improvement, and neuronal apoptosis reversal (see Table 1).

**Table 1:** Comparative overview of formulation and delivery technologies with PD-relevant readouts.

Technology	Representative system/carrier features	Lead active	Route	Key pharmacological/exposure improvements (summary)	Disease model	References
Nanoemulsion (O/W)	Porphyrin-assisted curcumin nanoemulsion	Curcumin (CUR)	Oral/i.p.	Improves motor performance; decreases malondialdehyde (MDA); increases superoxide dismutase (SOD) and glutathione peroxidase (GSH-Px) activities; stabilizes mitochondrial function compared with free curcumin	6-OHDA rats	(113)
Microemulsion	Oil-in-water microemulsion optimized by central composite design	Silymarin (SLM)	Intranasal	Increases brain distribution; improves motor performance; suppresses neuroinflammation and oxidative stress	MPTP rats	(114)
Biomimetic liposomes	NK-cell membrane-camouflaged CUR liposomes (BLIPO-CUR), meningeal lymphatic route	Curcumin (CUR)	MLVs-guided	Enables targeted delivery to dopaminergic neurons; enhances ROS scavenging; improves motor behavior; reduces neuronal apoptosis	PD mice	(115)

i.p., intraperitoneal; MDA, malondialdehyde; SOD, superoxide dismutase; GSH-Px, glutathione peroxidase; MLVs, meningeal lymphatic vessels.

#### 4.2.2 Dosage

Currently, dose optimization of TCM bioactives for PD is mainly based on *in vitro* and animal studies, with limited clinical validation. For instance, Tan et al. (34) treated PC12 cells with 6-OHDA (100 μM) and observed a dose-dependent increase in cell viability as APS concentration increased from 50 μM to 100 μM to 200 μM. Another study (29) in a silkworm PD model found that LBP at 50, 100, and 200 mg/kg doses improved motor function and dopamine levels in a dose-dependent manner. Zhu et al. (116) reported that in MPTP-induced PD mice, intraperitoneal CUR at low (40 mg/kg), medium (80 mg/kg), and high (160 mg/kg) doses resulted in dose-dependent improvements in motor function and dopaminergic neuron protection. Human-equivalent doses (HEDs) were estimated using standard allometric scaling [HED (mg/kg) = animal dose (mg/kg) × Km_animal/Km_human; Km_human = 37]. These values are illustrative anchors for hypothesis generation rather than clinical dose recommendations and must be refined by human PK/PD data. Preclinical doses are reported as originally published and should not be interpreted as clinical recommendations. Illustrative HEDs (allometric scaling; Km_mouse = 3, Km_rat = 6, Km_human = 37): CUR 80 mg/kg (mouse) → HED ≈ 80 × (3/37) = 6.5 mg/kg (~455 mg/day for 70 kg adult). CUR 160 mg/kg (mouse) → HED ≈ 13.0 mg/kg (~910 mg/day for 70 kg). LBP 100 mg/kg d (rat) → HED ≈ 100 × (6/37) = 16.2 mg/kg (~1.13 g/day for 70 kg).

### 4.3 Human evidence, pharmacokinetic limits, and trial design lessons

Human clinical evidence remains limited and underpowered. Across small, heterogeneous trials of curcumin and EGCG, outcomes are mixed and no disease-modifying efficacy has been established. Oral bioavailability and formulation choice cap target exposure for many MFH actives; for catechins, dose-related liver signals necessitate monitoring. Practical implications are: (i) exposure first—use high-exposure formulations with early PK/PD readouts and predefined target-engagement biomarkers (e.g., Nrf2 transcriptional signature, 4-HNE/MDA, iron/ferroptosis panels); (ii) design discipline—apply delayed-start, futility, or adaptive phase 2 frameworks with ≥24–52-week follow-up and co-primary endpoints pairing MDS-UPDRS with biomarker change; (iii) safety and interactions—embed liver-function monitoring for catechin-rich products and reconcile concomitant dopaminergic/MAO-B therapies. A side-by-side map of preclinical signals versus human constraints and actionable next steps appears in Table 2, with consolidated human evidence, PK ceilings, safety notes, and regulatory considerations detailed in Sections 6.3–6.5.

**Table 2:** Preclinical versus clinical evidence for major MFH bioactives in Parkinson’s disease: translational gaps and action items.

Bioactive (MFH)	Key preclinical PD findings (models/endpoints)	Clinical (PD) evidence overview	PK/Exposure constraints	Safety/Drug interactions	Critical translational gaps & action items
Curcumin (CUR)	Rotenone/MPTP models: activates Nrf2–ARE; upregulates HO-1/NQO1; inhibits microglial NLRP3; improves motor outcomes and TH^+^ neuron survival	Small, short-duration trials with mixed outcomes; formulation heterogeneity; no established disease-modifying benefit	Very low oral bioavailability; requires advanced delivery (nano/liposomes/intranasal) for adequate exposure	Generally well tolerated; monitor potential interactions with MAO-B inhibitors and polypharmacy	Use high-exposure formulations; pair MDS-UPDRS with target-engagement biomarkers (Nrf2 signature, 4-HNE/MDA); ≥24–52-week randomized designs
EGCG (green-tea catechin)	6-OHDA/MPTP/PFFs/*Drosophila*: enhances Nrf2 signaling; suppresses ferroptosis and neuroinflammation; improves behavior	Exploratory/small trials with inconsistent results; no proven efficacy	Limited exposure at tolerable oral doses; liver safety signal constrains high dosing	Require liver-function monitoring at higher intakes; potential interactions with L-dopa/MAO-B inhibitors	Define exposure–response; implement safety monitoring; biomarker-enriched enrollment targeting iron/lipid-peroxidation phenotypes
Ginsenoside Rg1 (G-Rg1)	Cell/MPTP: activates PI3K/Akt; promotes autophagy; facilitates α-synuclein clearance; neuroprotection	No positive, adequately powered PD RCTs; human PD data scarce	Oral absorption and brain delivery uncertain; delivery optimization needed	Generally favorable safety; interindividual variability expected	Conduct PK/brain-exposure and target-engagement PoC studies prior to efficacy trials
Glycyrrhizic acid (GA)	Inhibits HMGB1/TLR4/NF-κB; activates PI3K/Akt; improves motor and oxidative-stress readouts in MPTP models	PD clinical data lacking; human evidence mainly from other indications	Oral exposure feasible but variable	Watch for mineralocorticoid-like effects with chronic/high doses	Define therapeutic window; early PD PoC with inflammatory readouts and safety monitoring
Hesperidin (HSD)	6-OHDA/*Drosophila*: activates Nrf2–ARE; suppresses NF-κB; lowers MDA; improves motor function	No PD clinical trials with efficacy readouts	Exposure limited orally; formulation can enhance bioavailability	Generally well tolerated	Stratify by microbiome/inflammatory phenotype; evaluate food-format formulations in early trials
β-Carotene (β-Car)	*Drosophila* PD: increased SOD and GSH-Px activities; decreased ROS and MDA levels; improved motor function	Nutritional epidemiology suggests inverse associations with PD prevalence and all-cause mortality (non-causal)	Lipid solubility and dose/formulation strongly influence exposure	High-dose caution in smokers	Avoid causal inference from observational data; perform exposure–response bridging and safety window definition
*Lycium barbarum* polysaccharides (LBP)/*Astragalus* polysaccharides (APS)	Activate Nrf2–ARE; suppress NF-κB; support mitochondrial homeostasis; PD-adjacent/PD models show antioxidant and functional signals	No PD clinical evidence	Large molecules with poor oral absorption; consider intranasal or carrier-assisted delivery	Generally favorable safety profiles	Standardize structure–function specifications (MW/conformation); implement function-linked release tests (e.g., Nrf2 reporter)

### 4.4 Current limitations and future directions

Although existing data on dosage forms and preclinical dosing support the potential application of dietary supplements in PD, several challenges remain. First, the current evidence base is relatively weak, consisting primarily of *in vitro*, small-animal, or observational studies. Large-scale, multicenter, double-blind randomized controlled trials are needed to determine optimal dosing and safety profiles. Second, standardization of bioactive content remains difficult due to significant batch variation resulting from differences in geographical origin and processing methods, necessitating development of robust quality fingerprinting and regulatory standards (117, 118). Translation will require layered quality systems: (i) Identity/authenticity by DNA barcoding plus pharmacopeial macro-/microscopy; (ii) Marker quantification via HPLC/UPLC-UV or LC-MS/MS panels (e.g., curcuminoids for CUR; catechins including EGCG for tea extracts; Rg1/Rb1 for Panax; glycyrrhizin for Glycyrrhiza; HPSEC-MALS molecular-weight profiles for LBP/APS); (iii) Impurity limits (heavy metals, pesticides, residual solvents, mycotoxins, microbiology); (iv) Chemometric fingerprints (NIR/FT-IR with similarity indices) to manage geographical and processing variability; and (v) ICH stability programs to establish shelf-life. These align with cGMP and support reproducible clinical use. In addition to chemical fingerprints, include PD-relevant potency assays—e.g., Nrf2/ARE reporter activation, inhibition of NF-κB nuclear translocation in microglia, and survival of human iPSC-derived TH^+^ neurons under oxidative stress—to set acceptance criteria that bridge identity → content → function. Third, individual variability in therapeutic response due to genetic polymorphisms (119), gut microbiota composition (120), and concomitant medications (121) calls for development of personalized supplementation strategies under a precision nutrition framework. Finally, existing regulations limit the labeling claims of dietary supplements, precluding their promotion as “treatment” or “prevention” for PD (122, 123), which may influence clinician and patient acceptance (124). Long-term human safety and tolerability data specific to PD remain limited; consequently, adequately powered randomized trials with ≥24 to 52 weeks of follow-up are needed to define exposure–response and risk profiles. In summary, the absence of high-quality, large-scale randomized controlled trials in PD populations remains a key gap. Future research should prioritize clinical trial validation, multi-component synergy analysis, and regulatory alignment to enable the successful translation of dietary interventions into practical adjunctive management tools for PD. Incomplete reporting of design safeguards across studies precluded a definitive risk-of-bias determination; accordingly, our synthesis should be regarded as hypothesis-generating pending confirmatory trials.

Practical translational barriers include GMP-scale manufacturability, shelf-life stability under real-world storage, cost-of-goods and pricing, and consumer acceptability (sensory attributes, pill burden, dosing frequency). Regulatory constraints—spanning DSHEA/FDA/EFSA limits on disease claims, substantiation standards for structure–function statements, ingredient/novel-food authorization, raw-material identity testing, cGMP batch documentation, and post-market surveillance—may restrict labeling and clinical uptake in PD.

### 4.5 Safety, dosing, and regulatory checklist

*Dosing anchors*: Start from HED back-calculations yet must be refined by human PK/brain-exposure; avoid cross-route extrapolations (i.p. → oral). For data, see Table 3.

**Table 3:** Representative preclinical doses and illustrative human-equivalent dose (HED) anchors for key MFH actives.

Compound	PD model/context	Species	Route	Animal dose (mg/kg)	Estimated HED (mg/kg)	Evidence type	References
EGCG	α-synuclein pre-formed fibrils (PFF) model; 7-day pretreatment	Mouse (C57BL/6)	Oral	10	0.81	PD, mammalian	(53)
CUR	Rotenone-induced PD model	Mouse	Intraperitoneal	50	4.05	PD, mammalian	(48)
CUR	MPTP-induced PD model (dose–response)	Mouse	Intraperitoneal	40; 80; 160	3.24; 6.49; 12.97	PD, mammalian	(117)
LBP	CoCl₂-treated behavioral impairment model (indirect, non-PD)	Rat	Intraperitoneal	100	16.22	Indirect (non-PD), mammalian	(30)
HSD	6-OHDA-induced PD model (aged)	Rat	Oral	50	4.05	PD, mammalian	(61)

HED = animal dose × Km_animal/Km_human; Km_mouse = 3, Km_rat = 6, Km_human = 37. Values are anchors for hypothesis generation—not clinical recommendations. Several entries use intraperitoneal dosing in mice; translation to oral dietary-supplement design requires caution due to route-dependent bioavailability and brain exposure differences. These HED calculations are illustrative anchors for hypothesis generation only and are not clinical dosing recommendations.

*Safety monitoring*: Routine labs with hepatic panels for high-dose catechins; screen for herb–drug interactions with L-dopa/MAO-B inhibitors (transporters/MAO-B modulation).

*Formulation*: Prefer nano/micro-delivery or biomimetic liposomes with batch QC linking content → function (Nrf2, NF-κB, iPSC-TH^+^ assays).

*Labeling/regulatory*: Adhere to DSHEA/EFSA—structure–function claims only; prohibit disease-treatment claims; ensure identity/impurity/stability files.

*Trial-readiness*: Predefine pass/fail potency tied to QC assays; choose ≥24–52 week randomized designs with exposure–response analyses.

## 5 Conclusion and outlook

This review highlights recent advances in the study of polysaccharides, saponins/triterpenes, polyphenols, carotenoids, and aromatic phenylpropanoids derived from medicine–food homologous herbs in alleviating oxidative stress and neurodegeneration in Parkinson’s disease. Through multi-target actions (free-radical scavenging, inflammation suppression, autophagy activation, and mitochondrial support), these compounds show preclinical neuroprotective promise; no clinical efficacy has been established.

Given their synergistic potential, these TCM bioactives—when formulated as nanoemulsions, microemulsions, liposomes, or microencapsulation—can achieve improved bioavailability and brain permeability. This paves a feasible path for their integration into functional foods and dietary supplements. Although current dosage studies remain insufficient, future research conducted under GRAS and FDA/EFSA frameworks—focusing on dose–response relationships and long-term safety evaluations—will help ensure both therapeutic efficacy and patient adherence.

Moving forward, dietary supplement research should advance along two primary axes: (i) evidence-based and precision-guided strategies—pre-specify stratifiers (oxidative-stress and detoxification gene variants, baseline redox panels, and gut-microbiome profiles) and test treatment-by-biomarker interactions; and (ii) full-chain standardization and mechanistic decoding, including raw material authentication, active content quantification, formulation optimization, and quality fingerprinting, supported by network pharmacology, multi-omics, and systems biology to elucidate compound–target–pathway networks and guide formula refinement and novel target discovery. As a practical example, network pharmacology can first overlap PD oxidative-stress gene modules with target sets of MFH actives to nominate shortlists of 2–3 component pairs (e.g., Rg1 + EGCG, or APS + CUR) that co-cover Nrf2/ARE and PI3K/Akt while dampening NF-κB. A small, preclinical formulation screen can then titrate dose ratios and delivery types (e.g., nanoemulsion vs. liposome) in MPTP/6-OHDA models, with multi-omics readouts—glutathione/redox panels, 4-HNE/MDA, Nrf2 target genes, and phospho-Akt—used to confirm target engagement. Hits advance to PK/brain-exposure and HED anchoring to inform first-in-human dose-finding, thereby linking mechanism discovery to formulation design with translational checkpoints.

Through coordinated implementation of these strategies and interdisciplinary collaboration, the translation of medicine-food homologous TCM bioactives into regulated and industrialized functional foods or dietary supplements for PD can be accelerated—offering patients safer, more effective, and longer-lasting adjunctive therapeutic options.

## 6 Evidence capsules (updates)

This section was added to meet the reviewers’ requests for updates on PD-specific oxidative stress, genetic intersections, human evidence (including PK/safety), and translational quality checkpoints.

### 6.1 PD-specific features of oxidative stress

#### 6.1.1 Dopamine oxidation and quinone stress

Contemporary syntheses reinforce that dopamine auto-oxidation generates reactive o-quinones and H_2_O_2_that selectively injure substantia nigra neurons through protein modification, mitochondrial compromise, and redox cycling. These processes help explain nigral vulnerability beyond generic oxidative stress paradigms (125).

#### 6.1.2 Neuromelanin–iron catalysis

Recent imaging and tissue studies emphasize neuromelanin as an iron-interacting matrix that facilitates Fenton chemistry and lipid peroxidation. Advances in neuromelanin-sensitive MRI and analytical pathology strengthen the link between iron handling, neuromelanin load, and PD-specific oxidative damage (126).

#### 6.1.3 Complex I hypofunction stratification

Multi-cohort and patient-derived data show that a biologically meaningful subset of idiopathic PD exhibits mitochondrial complex I deficiency aligned with higher ROS burden and clinical heterogeneity, supporting mitochondria-linked oxidative stress as a disease-relevant—not merely generic—axis (127).

#### 6.1.4 Implication for MFH

These three nodes (dopamine quinones, neuromelanin–iron catalysis, complex I deficits) provide concrete mechanistic entry points for MFH bioactives that activate Nrf2, stabilize mitochondria, or buffer ferroptotic lipid peroxidation.

### 6.2 Genetic forms and oxidative cross-talk (PINK1/Parkin, DJ-1, LRRK2, GBA1)

#### 6.2.1 PINK1/Parkin mitophagy failure → ROS spillover

Loss-of-function along the PINK1–Parkin axis impairs clearance of damaged mitochondria, increasing ROS and sensitizing dopaminergic neurons to oxidative injury; recent overviews consolidate mechanistic and *in vivo* evidence for this link (128).

#### 6.2.2 DJ-1 as a redox-responsive chaperone

DJ-1 participates in antioxidant defense and proteostasis through cysteine-centered redox sensing; disease-linked variants undermine these functions and heighten oxidative vulnerability (129).

#### 6.2.3 LRRK2 and mitochondrial–inflammatory cross-talk

Elevated LRRK2 activity has been tied to mitochondrial DNA damage responses and inflammatory signaling, situating LRRK2 at the intersection of redox stress and innate immunity in PD-relevant systems (130).

#### 6.2.4 GBA1 and lysosomal flux

Glucocerebrosidase deficiency perturbs lysosomal clearance and lipid homeostasis, secondarily amplifying oxidative stress and mitochondrial dysfunction; recent work highlights GCase–mitochondria interactions in PD pathobiology (131).

#### 6.2.5 Implication for MFH

These genetic axes motivate hypotheses that MFH actives could: (i) support mitophagy/mitochondrial quality control, (ii) reinforce endogenous redox buffering (e.g., Nrf2-GPX4), and (iii) dampen inflammation linked to mitochondrial stress.

### 6.3 Human evidence map, pharmacokinetic constraints, and safety

See Table 2 for a consolidated grid.

#### 6.3.1 Efficacy signals

Human studies of canonical MFH-relevant polyphenols (e.g., curcumin, EGCG) remain small and methodologically limited; no disease-modifying efficacy has been established in PD. Concordant reviews conclude that heterogeneous designs, short durations, and insufficient target engagement preclude firm conclusions (132).

#### 6.3.2 PK ceiling and exposure limits

Oral bioavailability is a principal barrier for curcumin and several flavonoids; for green-tea catechins, regulatory toxicology summaries highlight dose-related liver signals that cap safe exposure and necessitate liver-function monitoring in products and trials (133).

#### 6.3.3 Trial-design lessons

Future trials should:

enrich for participants with high redox/ferroptosis burden (biomarker-enriched enrollment);
use delayed-start or target-engagement frameworks;
pair clinical trajectories (e.g., MDS-UPDRS) with biochemical readouts (Nrf2 transcriptional signature, MDA/4-HNE, iron/ferroptosis panels) to confirm on-target exposure.

### 6.4 Drug–nutrient interactions and practical dosing considerations

Catechin-rich products and other phenolic MFH actives can interact with drug-metabolizing enzymes and transporters; vigilance is warranted when co-administered with dopaminergic agents or MAO-B inhibitors. Current PD-specific clinical interaction data are sparse; risk management should include medication reconciliation and—when high-dose catechins are contemplated—liver-function monitoring in line with regulatory guidance (134).

### 6.5 Translational quality and regulatory checkpoints

#### 6.5.1 Non-targeted fingerprinting + chemometrics

2024 updates advocate untargeted spectral fingerprints (e.g., NIR/FT-IR/LC-UV) plus chemometrics to manage geo-processing variability, detect adulteration, and ensure batch-to-batch comparability in complex MFH matrices (135).

#### 6.5.2 Polysaccharide structure–function alignment

Systematic reviews emphasize the centrality of molecular weight and conformation to the bioactivity of medicinal polysaccharides, recommending advanced sizing/characterization (e.g., HPSEC-MALS) for specification setting and post-change control (136).

#### 6.5.3 Function-linked release tests

To bridge identity → content → function, MFH products intended for PD research should adopt mechanism-linked release assays (e.g., Nrf2/ARE reporter activation, NF-κB nuclear-translocation inhibition in microglia, protection of human iPSC-derived TH^+^ neurons under oxidative stress) in addition to pharmacopeial quality tests (see Table 4 in the main text).

**Table 4:** Quality control and standardization from raw material to finished dosage form: an operational checklist.

Layer	Specific measures/methods	Key outputs/acceptance criteria	Notes/illustrative references
Identity/authenticity	DNA barcoding; pharmacopeial macro-/microscopy	Correct species/part; exclusion of adulterants/substitutes	Supports MFH source verification (119)
Marker quantification	HPLC/UPLC-UV or LC-MS/MS panels (e.g., curcuminoids; catechins incl. EGCG; Rg1/Rb1; glycyrrhizin; HPSEC-MALS for LBP/APS MW profiles)	Target ranges with inter-batch RSD controls	Links to formulation ratios and label claims (119)
Impurity limits	Heavy metals, pesticides, residual solvents, mycotoxins, microbiology	Meets dietary-supplement limits	Harmonize with DSHEA/EFSA & cGMP release (123, 124)
Fingerprinting/chemometrics	NIR/FT-IR/LC-UV fingerprints with similarity indices	Geographic/processing variability controlled; anomaly detection	Batch-to-batch comparability (119)
Stability	ICH long-term/accelerated (T/RH/light)	Shelf-life and storage conditions established	Track both actives and dosage form attributes (119)
Functional release (PD-relevant)	Nrf2/ARE reporter activation; reduced NF-κB nuclear translocation; improved survival of human iPSC-derived TH^+^ neurons under oxidative stress; meeting prespecified pass/fail potency thresholds tied to content	Prespecified pass/fail potency thresholds tied to content	Mechanism-linked release; neural relevance (84–87, 91–95)

MFH, medicine–food homology; HPSEC-MALS, high-performance size-exclusion chromatography with multi-angle light scattering; TH^+^, tyrosine hydroxylase-positive; T/RH, temperature/relative humidity; DSHEA, Dietary Supplement Health and Education Act.

### 6.6 Anchors to the mechanism sections: ferroptosis and the gut–brain axis

#### 6.6.1 Ferroptosis–Nrf2 intersection

Recent PD-focused syntheses place GPX4 activity, iron handling, and lipid peroxidation at the core of ferroptotic vulnerability. Nrf2 activation converges on these nodes (e.g., iron sequestration, glutathione metabolism), supporting the cross-talk summarized in Section 3.1 and Figure 1 (137).

#### 6.6.2 Gut–brain axis

Contemporary reviews integrate PD-associated dysbiosis with oxidative–inflammatory signaling and discuss how dietary polyphenols and polysaccharides can modulate microbiota, barrier function, and downstream neuroinflammation, aligning with the “oxidative–inflammatory–gut” thread highlighted in Sections 3.3 and 4.4 (138).

### 6.7 Epidemiology capsule (2024–2025 updates)

#### 6.7.1 Global projections

A 2025 BMJ modeling study based on GBD 2021 projects 25.2 million people living with PD by 2050 (≈ + 112% from 2021), with a global prevalence of ~267/100,000 in 2050 and regional heterogeneity; most growth is attributable to population aging and growth (3).

#### 6.7.2 Systematic prevalence evidence

A 2024 meta-analysis reports substantial between-region differences in PD prevalence (highest point estimates in East Asia & Pacific), underscoring how study design and case-ascertainment influence pooled estimates (139).

#### 6.7.3 Authoritative overviews

2024 narrative syntheses highlight that prevalence has risen beyond demographic change alone, while incidence trends remain uncertain in many regions due to data quality gaps (140).

#### 6.7.4 Regional updates (Asia)

A 2024 regional burden analysis (1990–2021) shows marked increases in incidence (+198%), prevalence (+284%), mortality (+111%), and DALYs (+144%) across Asia, emphasizing geographic variability relevant to projections (141).

#### 6.7.5 Temporal trend reassessments

Methodologically updated 2024 analyses suggest overall PD prevalence ≈3.15/1,000, notably exceeding older pooled estimates, again reflecting differing methods and ascertainment (142).

#### 6.7.6 Implication for Section 1.1

These multi-source data triangulate the message that PD prevalence is rising but absolute estimates vary with modeling choices, case definitions, and regional data quality—hence our choice in Section 1.1 to cite multiple recent sources rather than a single model.

## Glossary

PD: Parkinson’s disease
SNpc: Substantia nigra pars compacta
Nrf2/ARE: Nuclear factor erythroid 2–related factor 2/antioxidant response element
NF-κB: Nuclear factor kappa-light-chain-enhancer of activated B cells
MMP: Mitochondrial membrane potential
SOD: Superoxide dismutase
GSH-Px: Glutathione peroxidase
ROS: Reactive oxygen species
PC12: Pheochromocytoma cell line PC12
SH-SY5Y: Human neuroblastoma cell line SH-SY5Y
MPTP: 1-Methyl-4-phenyl-1,2,3,6-tetrahydropyridine
6-OHDA: 6-Hydroxydopamine
HO-1: Heme oxygenase-1
NQO1: NAD(P)H quinone dehydrogenase 1
PI3K/Akt: Phosphoinositide 3-kinase/protein kinase B
AMPK: AMP-activated protein kinase
mTOR: Mechanistic target of rapamycin
MDA: Malondialdehyde
TLR4: Toll-like receptor 4
MyD88: Myeloid differentiation primary response protein 88
HMGB1: High mobility group box 1
Bcl-2: B-cell lymphoma 2
Bax: Bcl-2-associated X protein
MAPK: Mitogen-activated protein kinase
TNF-α: Tumor necrosis factor-alpha
IL-1β: Interleukin-1 beta
IL-6: Interleukin-6
MAO-B: Monoamine oxidase B
COX-2: Cyclooxygenase-2
LBP: *Lycium barbarum* polysaccharides
APS: *Astragalus* polysaccharides
G-Rg1: Ginsenoside Rg1
GA: Glycyrrhizic acid
CUR: Curcumin
EGCG: Epigallocatechin gallate
PFFs: Pre-formed fibrils
CAT: Catalase
GST: Glutathione S-transferase
PCP: Pachymaran
GLTs: Ganoderma triterpenes
HF: Hawthorn flavonoids
PINK1: PTEN-induced putative kinase 1
MDS-UPDRS: Movement Disorder Society-Unified Parkinson’s Disease Rating Scale
PDQ-39: Parkinson’s Disease Questionnaire-39
GRAS: Generally Recognized as Safe
FDA: Food and Drug Administration
EFSA: European Food Safety Authority
RCT: Randomized controlled trial
TCM: Traditional Chinese Medicine
