# Supplementary material for: Medicine–food homology bioactives in Parkinson’s disease: multi-target oxidative-stress modulation and translation to dietary supplements
Source: Front Nutr. 2025 Nov 21;12:1677749. doi: 10.3389/fnut.2025.1677749 (PMC12678100; doi:10.3389/fnut.2025.1677749)
Supplement: Supplementary file 1 [file Table_1.docx]

Appendix A:

| Database | Interface/Platform | Coverage Window | Last Search Date | Language Filter | Concept Blocks (one-line summary) | Notes |
| --- | --- | --- | --- | --- | --- | --- |
| PubMed | NLM | Inception–Aug 1, 2025 | 1-Aug-25 | English | Parkinson’s disease AND (oxidative stress OR Nrf2/ARE OR NF-κB OR PI3K/Akt OR autophagy) AND (medicinal-food homologous TCM terms: polysaccharide, saponin/triterpene, polyphenol, carotenoid; exemplar compounds/herbs) | Includes in vitro, invertebrate, and PD-specific rodent models; excludes non-PD basic unless mechanistically translational. |
| Web of Science Core Collection | Clarivate | Inception–Aug 1, 2025 | 1-Aug-25 | English | Same concept blocks; TS/AB/Title fields combined with Boolean operators | Science Citation Index Expanded prioritized. |
| Embase (Ovid) | Ovid | Inception–Aug 1, 2025 | 1-Aug-25 | English | Same concept blocks; Emtree terms mapped to free-text | De-duplicated against MEDLINE where applicable. |
| Cochrane Library | Wiley | Inception–Aug 1, 2025 | 1-Aug-25 | English | Same concept blocks in CENTRAL/Reviews | Used to identify trials and mechanistic reviews. |

Scope and eligibility. Primary in vitro, invertebrate, and PD-specific rodent studies; epidemiologic analyses; and formulation/delivery reports related to oxidative stress in PD were eligible. Narrative reviews and non-PD basic studies were excluded unless they provided translational mechanistic rationale.

Search strategy. Database-specific Boolean strategies, field tags, truncations, and controlled vocabulary mappings (MeSH/Emtree) are provided below verbatim.

Limitations. This was a structured narrative synthesis without quantitative meta-analysis or tool-based risk-of-bias scoring; we qualitatively noted reported design safeguards. Most included records were published between 2020 and 2025, reflecting field growth and potentially introducing recency/publication biases.

PubMed:(("Parkinson Disease"[Mesh] OR "Parkinson's disease"[tiab] OR "Parkinson disease"[tiab])

AND ("Oxidative Stress"[Mesh] OR "oxidative stress"[tiab] OR Nrf2[tiab] OR NFE2L2[tiab] OR "antioxidant response element"[tiab] OR "NF-kappa B"[tiab] OR "NF-κB"[tiab] OR "PI3K/Akt"[tiab] OR autophag*[tiab]) AND (polysaccharid*[tiab] OR saponin*[tiab] OR triterpen*[tiab] OR polyphenol*[tiab] OR carotenoid*[tiab] OR eugenol[tiab] OR hesperidin[tiab] OR hesperetin[tiab] OR curcumin[tiab] OR "epigallocatechin gallate"[tiab] OR EGCG[tiab] OR Lycium[tiab] OR "Lycium barbarum"[tiab] OR "Poria cocos"[tiab] OR "Wolfiporia extensa"[tiab] OR Panax[tiab] OR "ginsenoside Rg1"[tiab] OR Glycyrrhiza[tiab] OR "Glycyrrhiza uralensis"[tiab] OR Citrus[tiab] OR "Citrus reticulata"[tiab] OR "medicine-food homology"[tiab] OR "medicinal and food homology"[tiab])) AND (English[lang])

Embase:('parkinson disease'/exp OR 'parkinson disease':ti,ab OR "parkinson's disease":ti,ab) AND ('oxidative stress'/exp OR 'oxidative stress':ti,ab OR nrf2:ti,ab OR nfe2l2:ti,ab OR 'antioxidant response element':ti,ab OR 'nf kappa b':ti,ab OR 'NF-κB':ti,ab OR 'pi3k/akt':ti,ab OR autophag*:ti,ab) AND (polysaccharid*:ti,ab OR saponin*:ti,ab OR triterpen*:ti,ab OR polyphenol*:ti,ab OR carotenoid*:ti,ab OR eugenol:ti,ab OR hesperidin:ti,ab OR hesperetin:ti,ab OR curcumin:ti,ab OR 'epigallocatechin gallate':ti,ab OR egcg:ti,ab OR lycium:ti,ab OR 'lycium barbarum':ti,ab OR 'poria cocos':ti,ab OR 'wolfiporia extensa':ti,ab OR panax:ti,ab OR 'ginsenoside rg1':ti,ab OR glycyrrhiza:ti,ab OR 'glycyrrhiza uralensis':ti,ab OR citrus:ti,ab OR 'citrus reticulata':ti,ab OR 'medicine-food homology':ti,ab OR 'medicinal and food homology':ti,ab) AND [english]/lim

Web of Science Core Collection:TS=( (("Parkinson" NEAR/1 disease) OR "Parkinson's disease") AND ("oxidative stress" OR Nrf2 OR NFE2L2 OR "antioxidant response element" OR "NF-κB" OR "NF-kappa B" OR "PI3K/Akt" OR autophag*) AND (polysaccharid* OR saponin* OR triterpen* OR polyphenol* OR carotenoid* OR eugenol OR hesperidin OR hesperetin OR curcumin OR "epigallocatechin gallate" OR EGCG OR Lycium OR "Lycium barbarum" OR "Poria cocos" OR "Wolfiporia extensa" OR Panax OR "ginsenoside Rg1" OR Glycyrrhiza OR "Glycyrrhiza uralensis" OR Citrus OR "Citrus reticulata" OR "medicine-food homology" OR "medicinal and food homology")) AND LANGUAGE: (English)

Cochrane Library:(("Parkinson's disease":ti,ab,kw OR (Parkinson NEXT disease):ti,ab,kw) AND ("oxidative stress":ti,ab,kw OR Nrf2:ti,ab,kw OR NFE2L2:ti,ab,kw OR "antioxidant response element":ti,ab,kw OR "NF-kappa B":ti,ab,kw OR "NF-κB":ti,ab,kw OR "PI3K/Akt":ti,ab,kw OR autophag*:ti,ab,kw) AND (polysaccharid*:ti,ab,kw OR saponin*:ti,ab,kw OR triterpen*:ti,ab,kw OR polyphenol*:ti,ab,kw OR carotenoid*:ti,ab,kw OR eugenol:ti,ab,kw OR hesperidin:ti,ab,kw OR hesperetin:ti,ab,kw OR curcumin:ti,ab,kw OR "epigallocatechin gallate":ti,ab,kw OR EGCG:ti,ab,kw OR Lycium:ti,ab,kw OR "Lycium barbarum":ti,ab,kw OR "Poria cocos":ti,ab,kw OR "Wolfiporia extensa":ti,ab,kw OR Panax:ti,ab,kw OR "ginsenoside Rg1":ti,ab,kw OR Glycyrrhiza:ti,ab,kw OR "Glycyrrhiza uralensis":ti,ab,kw OR Citrus:ti,ab,kw OR "Citrus reticulata":ti,ab,kw OR "medicine-food homology":ti,ab,kw OR "medicinal and food homology":ti,ab,kw))

General note: No lower date bound (database inception to August 1, 2025); English-language filter applied; no species/age limits; study-type filters not applied beyond eligibility rules specified in the main text. During screening we prioritized records published in the preceding five years (January 1, 2020–August 1, 2025) to emphasize contemporaneous evidence; older seminal studies were included when essential for mechanistic or historical context.

Appendix B:

**Supplementary Table 1.** Representative MFH Active Constituents Relevant to Parkinson’s Disease: Compound-to-Pathway Mapping for Oxidative-Stress Mechanisms

**Columns:** class (polysaccharides, saponins/triterpenes, polyphenols, carotenoids, phenylpropanoids), active constituent, primary PD-relevant mechanisms (e.g., Nrf2/ARE activation, NF-κB suppression, PI3K/Akt pro-survival signaling, autophagy promotion, mitochondrial stabilization), representative references. Note: Mechanistic entries may include supportive non-PD models as indicated in the main text.

| Category | Active Constituent | Mechanism in Parkinson’s Disease | References |
| --- | --- | --- | --- |
| Polysaccharides | Lycium barbarum Polysaccharides (LBP) | Activates the Nrf2/ARE antioxidant pathway—evidenced by increased SOD and GSH-Px activity, enhanced ROS scavenging, and stabilization of the mitochondrial membrane potential—and inhibits NF-κB signaling by reducing IκBα phosphorylation and p65 nuclear translocation and by lowering TNF-α and IL-1β. | (24, 25, 26, 27, 28) |
|  | Astragalus Polysaccharides (APS) | Alleviates oxidative stress via Nrf2/ARE activation and inhibition of TLR4/MyD88/NF-κB signaling (increases SOD and GSH-Px; decreases MDA) and activates PI3K/Akt (raises the Bcl-2/Bax ratio; reduces caspase-3 activation). | (23, 33, 34) |
| Saponins/Triterpenes | Ginsenoside Rg1 (G‑Rg1) | Enhances neuronal survival through PI3K/Akt anti-apoptotic signaling (increases Bcl-2; decreases Bad; reduces caspase-3 activation) and promotes autophagy via AMPK activation with concomitant mTOR inhibition (elevates Beclin-1 and LC3-II; activates Nrf2). | (35, 36, 37) |
|  | Glycyrrhizic Acid (GA) | Inhibits HMGB1 release and downstream TLR4/NF-κB signaling and activates PI3K/Akt, increasing the Bcl-2/Bax ratio, reducing caspase-3 activation, and elevating reduced glutathione (GSH) to preserve mitochondrial function and attenuate inflammation. | (40, 41, 42, 43) |
| Polyphenols | Curcumin (CUR) | Reversibly inhibits monoamine oxidase-B (MAO-B), activates Nrf2/ARE signaling (upregulates HO-1 and NQO1), and inhibits NF-κB signaling (reduces p65 nuclear translocation; decreases COX-2 and IL-6 expression). | (44, 45, 46) |
|  | Epigallocatechin Gallate (EGCG) | Scavenges reactive oxygen species (ROS), activates the p62–Keap1–Nrf2/ARE axis, and inhibits NF-κB signaling by reducing p65 nuclear translocation and decreasing COX-2 and IL-6 expression. | (49, 50, 51) |
| Aromatic phenylpropanoids | Eugenol | Restores SOD and GSH-Px activities and GSH levels, decreases malondialdehyde (MDA), and mitigates oxidative damage; co-administration with levodopa further increases GSH. | (56, 57, 58) |
|  | Hesperidin (HSD) | Activates Nrf2/ARE signaling (upregulates HO-1 and NQO1) and inhibits NF-κB signaling (reduces IκBα phosphorylation and p65 nuclear translocation), promoting ROS clearance and preserving mitochondrial function. | (59, 60) |
| Carotenoids | β‑Carotene (β‑Car) | Decreases MDA and NF-κB p65 nuclear translocation, increases SOD, GSH, and GSH-Px, and improves motor function while supporting dopaminergic (TH⁺) neuron survival. | (63, 64) |
|  | Lutein | Promotes Nrf2 nuclear translocation with HO-1 and NQO1 upregulation and suppresses NF-κB signaling by reducing p65 nuclear translocation and decreasing TNF-α and IL-1β, thereby enhancing ROS clearance and mitigating neuroinflammation. | (67, 68) |

**Supplementary Table 2.** Pathway-Level Mapping of MFH Active Constituents Relevant to Parkinson’s Disease

**Columns:** pathway (Nrf2/ARE activation; PI3K/Akt activation; NF-κB suppression); representative active constituents (e.g., LBP, APS, CUR, EGCG, G-Rg1, GA, HSD); principal PD-relevant actions (Nrf2 nuclear translocation and HO-1/NQO1 upregulation; increased Bcl-2/Bax ratio; reduced caspase-3 activation; inhibited IκBα phosphorylation and p65 nuclear translocation); references. Note: Mechanistic entries are derived primarily from PD-relevant models; supportive non-PD evidence, where cited, is indicated in the main text.

| Pathway | Active Constituents | Application | References |
| --- | --- | --- | --- |
| Nrf2/ARE Pathway Activation | LBP; APS; CUR; EGCG | Promotes Nrf2 nuclear translocation, upregulates HO‑1 and NQO1, enhances ROS clearance, restores SOD/GSH‑Px activity, and stabilizes mitochondrial membrane potential in PD models | (24, 33, 47, 52) |
| PI3K/Akt Signaling Modulation | G‑Rg1; APS; GA; EGCG | Activates PI3K/Akt–mTOR signaling to phosphorylate Akt, increase Bcl‑2/Bax ratio, inhibit caspase‑3, and enhance autophagy, thereby protecting dopaminergic neurons | (23, 34, 35, 36, 40, 54) |
| NF‑κB–Mediated Inflammation Suppression | LBP; EGCG; HSD; GA; CUR | Inhibits IκBα phosphorylation and p65 nuclear translocation, downregulates pro‑inflammatory cytokines (TNF‑α, IL‑1β, IL‑6, COX‑2), reducing neuroinflammation and improving behavior | (31, 43, 48, 55, 60) |
